# Supplementary material for: Development of chemokine network inhibitors using combinatorial saturation mutagenesis
Source: Commun Biol. 2025 Apr 3;8:549. doi: 10.1038/s42003-025-07778-6 (PMC11969024; doi:10.1038/s42003-025-07778-6)
Supplement: Supplementary file 4 — Supplementary Data 2 [file 42003_2025_7778_MOESM4_ESM.zip › SupplementaryData3/scripts/xattune_dr.tar!/./script/analysis/JK/JK_E21_CCL14_THP1_plot.pdf]

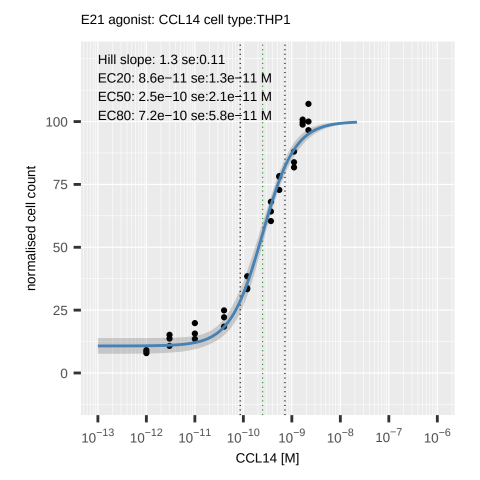
E21 agonist: CCL14 cell type:THP1
Hill slope: 1.3 se:0.11
EC20: 8.6e−11 se:1.3e−11 M
EC50: 2.5e−10 se:2.1e−11 M
EC80: 7.2e−10 se:5.8e−11 M
normalised cell count
100
75
50
25
0
10^{-13} 10^{-12} 10^{-11} 10^{-10} 10^{-9} 10^{-8} 10^{-7} 10^{-6}
CCL14 [M]
